# A Conceptual System of Antecedents and Processes in Social Entrepreneurship Opportunity Identification

**DOI:** 10.3389/fpsyg.2021.698892

**Published:** 2021-10-13

**Authors:** Virginie Vial, Katia Richomme-Huet

**Affiliations:** ^1^Strategy, Sustainable Development and Entrepreneurship Department, KEDGE Business School, Marseille, France; ^2^UMR 5062, Institut d’Asie Orientale (IAO), ENS de Lyon, Lyon, France

**Keywords:** Ashoka, empirical triangulation, grounded theory, inductive analysis, life stories, opportunity identification, semi-computerized content analysis, social entrepreneurship

## Abstract

Aiming to complement and ground the theory of social entrepreneurship opportunity identification, we draw from a database of 2,872 entrepreneurs’ life stories with two main objectives. The first is to provide a comprehensive list and categorization of antecedents of opportunity identification in the context of social entrepreneurship. The second is to demonstrate the systemic interconnections between those and build a model of social entrepreneurship opportunity identification. We review the literature and establish a framework of five high-order key antecedents’ areas (context, background, social networks and interactions, affect, and cognitive process). We then proceed to a five-step empirical triangulation methodology mixing computerized and manual content analysis. We thereby identify 42 antecedents nested into 17 first-level items grouped into the five high-order key antecedents’ areas. Our detailed results shed light on a wide array of previously ignored antecedents and provide more precisions about those that had already been documented elsewhere. Finally, we highlight and explain the relationships between the antecedents, show that they constitute an “opportunity growing ground,” and present a full model of social entrepreneurship opportunity identification based on their interconnections. The context of the social entrepreneur combines stable features regarding access to various resources, a strong geographical identity and history, the encounter of several worlds, all condition or are conditioned by his/her social networks and background. This context is also subject to diverse constraints and institutional barriers that can shape the entrepreneur’s background, her/his experiences, as well as his/her affect specificities. This stable context is at some point hit by elements of change that disrupt this stability, triggering chains of reactions between the various antecedents of opportunity identification.

## Introduction

“In a society that has chronically higher levels of violence and suicide rates than any other OECD countries, [social entrepreneur] Hye-Shin Chung, is enabling ordinary citizens to help themselves and people around them overcome their emotional and psychological trauma.” Over the past 60 years, a series of collective trauma deeply marked the Korean historical and socio-economic context. The population endured the war in the 50s, a military dictatorship in the 70s–80s, and suffered from a massive economic restructuring in response to the 1997 financial crisis. More recently, though less dramatic, the national tragedy of the Sewol Ferry Disaster in 2014 still “severely shocked Korean society” (Woo et al., 2015, p. 10,974), while painfully underlining government incompetence in dealing with the catastrophe. In the meantime, the Korean society is subject to intense pressure to excel at all times. Generating significant social consequences, prevention, classical medical treatment and professional consultation fail to address the resulting nation-wide mental and physical health issues.

Social entrepreneur Hye-Shin indeed “started seeing gaps in the profession” while building herself an educational and professional background in psychiatry, a path she embraced after being affected by the painful loss of her mother when she was only 11. In her first years as a professional, “she learned about the outpatient treatment services abroad for people with schizophrenia,” and implemented it in Korea through her private practice. Official institutions approved of this initiative and “major university hospitals started outpatient services.” Her “understanding deepened through her work” with Dong-won Park, a patient who - along with 20 other of his family members and over a 20-year period, had been falsely accused of espionage, tortured and imprisoned by state authorities. Through him and, later, “from her own experience working with victims and their family members,” she understood the interrelatedness of individual circumstances and collective experiences on one’s psychological health. Thanks to her practice, “she was convinced that empathy is a key” and that “those who suffered extreme emotional trauma (…) can be excellent counselors and healers (…) because they truly understand the pain and the process of recovery.” She then created “a set of highly replicable and self-multiplying approaches” for both collective and individual counseling. These take the form of a self-evaluation tool (‘Mind Report’) or empathy-based healing programs (‘Wounded Healers’ and ‘Everyone Needs a Mom’ programs) “to turn ordinary citizens into healing agents for themselves and others.” She also launched for-profit (‘Mind Prism’), as well as citizen-sector organizations (The Truth Foundation, The Warak Center and the Empathetic Person) “to empower more wounded healers from all walks of life” (laid-off workers, sexual minorities, disaster’s victims and families as well as ordinary citizens).

Hye-Shin’s life story vividly provides one example of the ‘why’ and the ‘how’ social entrepreneurs identify opportunities. In a specific national context, she spotted several gaps in the area of mental health treatment thanks to her personal, emotional, educational, and professional background and experience, which has been strengthened by her interaction with various patients, colleagues, and other stakeholders. These elements, combined with her exposure to alternative foreign health systems, helped her find opportunities and design pioneering social solutions. Her story highlights that a social enterprise presents some of the broad antecedents of opportunity identification (hereafter OI) that appear in the mainstream entrepreneurship literature – the context, background, social networks, affect and cognitive process (Venkataraman, 1997; Ardichvili et al., 2003; Gielnik et al., 2014). Can we detail those broad antecedents? What are the specificities of those antecedents and are there additional antecedents for the case of social entrepreneurship? For example, is the context consistently hostile (repeated collective traumas, failing institutions)? Are there backgrounds that are recurrent in social entrepreneurship (hereafter SE), and what are they (perseverance, solidarity)? What kind of social networks matter (informal citizen networks, professional networks)? What are the types of affect at play (empathy and grief)? How does the cognitive process lead to the OI (sensitivity to the other’s suffering, establishing institutional and practice gaps from exposure to various professional experiences)? There are indeed differences in the identification of social and business opportunities which call for a more complete and grounded theory of social OI (González et al., 2017).

While the force of a case study resides in its breadth of details, its weakness lies in its singularity (such as exceptional circumstances, out of the ordinary personalities, or very specific settings). As we are aiming for generalization, we need to expand and deepen the analysis regarding the qualification of each broad set of antecedents using a large collection of detailed cases. Such a collection is freely available from the world largest international network of social entrepreneurs, Ashoka^1^, counting 2,872 social entrepreneurs who have been elected Ashoka fellows between 1982 and 2016. We proceed by triangulation (Wilson, 1981), first adopting a quantitative approach by way of a computerized content analysis, and second proceeding to a qualitative discourse analysis. Using triangulation allows to “take advantage of both the qualitative and quantitative perspectives on texts, assuming that both the different forms of repetition and the peculiar way by which something has been said may play an important role” (Cortini and Tria, 2014, p. 561). We analyze the textual data with two objectives in mind. First, we target the development of a comprehensive categorization of antecedents of OI accounting for the specificities of SE opportunities. Second, we wish to expose the systemic relationships among the antecedents of OI to construct a conceptual model of social entrepreneurship opportunity identification (hereafter SEOI).

Through the thorough analysis of the rich social entrepreneurs’ life stories, we first offer a detailed and finely grained map of 42 opportunity identification antecedents nested into 17 first-level items grouped into the 5 classic generic antecedents. The comprehensive and dynamic analysis shows that these antecedents form an ensemble and web of relationships, which we coin the ‘Opportunity Growing Ground.’ We characterize relationships between antecedents to depict a full conceptual model of Social Entrepreneurship Opportunity Identification (SEOI). We hence provide the first comprehensive conceptual model of SEOI that is backed up by a solid empirical investigation.

The first section reviews the mainstream as well as social entrepreneurship literature so as to establish OI antecedents. The second section presents the data, explains and justifies the empirical methodology. The third section reports and discusses the results that are emerging from our inductive analysis. The last section concludes.

## Opportunity Identification

We first review the mainstream entrepreneurship literature to determine the generic OI antecedents. We then survey the literature that investigates the specificities of generic OI in social entrepreneurship. The literature review constitutes our point of departure for the analysis that follows.

### Opportunity Identification in Mainstream Entrepreneurship

Covering recognition, discovery and creation (Sarasvathy et al., 2003), OI represents the encounter of market supply and demand, which transforms into the creation of economic value. A central element in entrepreneurship research (Filser et al., 2020), the opportunity is instrumental to the understanding of the market equilibrium process (Klein, 2008). In effect, an opportunity is a way for the market to clear, a match between market supply and demand at the equilibrium price (Kirzner, 1979). Entrepreneurial opportunities are always described as profit opportunities (Foss et al., 2008; Klein, 2008), i.e., as “those situations in which new goods, services, raw materials, and organizing methods can be introduced and sold at greater than their cost of production” (Shane and Venkataraman, 2000, p. 220).

In a general perspective, original theories (Kirzner, 1973, 1979; Shane and Venkataraman, 2000; Stevenson and Jarillo, 2007), show that OI represents one of the most distinctive and fundamental entrepreneurial behaviors (Gaglio and Katz, 2001). The study of the antecedents of OI (Gielnik et al., 2014) may shed some light on both the “why” and the “how” some individuals do identify opportunities while others do not (Venkataraman, 1997; Tang et al., 2012; Busenitz et al., 2014).

(1) The reading of the literature reveals that individuals can identify entrepreneurial opportunities for several reasons:

(a) They possess a certain background encompassing personality traits and cognitive properties. These include self-efficacy, risk-taking, optimism and creativity, prior knowledge and experience (Bandura, 1986; Ajzen, 1987; Hills et al., 1997, 1999; Krueger, 2000; Ardichvili et al., 2003; Shane, 2003; Ko and Butler, 2007; Lehner and Kansikas, 2012; Grégoire and Shepherd, 2015).
(b) They are part of various social networks (Aldrich and Zimmer, 1986; Allen, 2000; Johannisson, 2000; Ardichvili et al., 2003; Shane, 2003; Ozgen and Baron, 2007; Grégoire and Shepherd, 2015).
(c) They experience different types of positive and negative affect (Baron, 2008; Cardon et al., 2012; Tang et al., 2012; Welpe et al., 2012; Grégoire, 2014; Foo et al., 2015; Grégoire and Shepherd, 2015; Zhao and Xie, 2020).
(d) They interact with their exogenous social, economic, technological and institutional context (Krueger, 2000; Baron, 2008; Grégoire and Shepherd, 2015).

(2) They go through a cognitive process:

(a) Mental models ordered to optimizing effectiveness within given situations, such as entrepreneurial alertness (Hu et al., 2018; Neneh, 2019; Li et al., 2020);
(b) Ability to notice business opportunities, and entrepreneurial intention (Li et al., 2020);
(c) Efforts made to achieve an entrepreneurial activity (Kirzner, 1973, 1997; Ajzen, 1987; Krueger, 2000; Gaglio and Katz, 2001; Ardichvili et al., 2003; Shook et al., 2003; Gaglio, 2004; McMullen and Shepherd, 2006; Mitchell et al., 2007; Foo, 2011; Gielnik et al., 2012; Tang et al., 2012; Foo et al., 2015; Grégoire and Shepherd, 2015; Brännback and Carsrud, 2016; Zhao and Xie, 2020).

In essence, the entrepreneurial OI results from the entrepreneur’s ‘cognitive process’ (the “how”) that feeds from the interactions of the entrepreneur’s ‘background,’ ‘affect,’ ‘social networks,’ and ‘context’ (the “why”). These represent the high-order key antecedents’ areas (hereafter HOKAAs) of OI that have readily been presented in the literature, and which we use as a generic grid of analysis in what follows.

### Opportunity Identification in Social Entrepreneurship

Social entrepreneurship opportunity identification antecedents are likely to present some specificities because they differ regarding:

(1) The target outcome: general human welfare versus sole economic value creation (Ruskin et al., 2016; Lambrechts et al., 2020);
(2) The beneficiaries: a variety of beneficiaries versus owners;
(3) The situation: the social venture is “placed between civil-society, the state and the market” (Lehner and Kansikas, 2012, p. 27) rather than solely on the market.

A narrower sphere of entrepreneurship but embracing a larger scope of opportunities, “social entrepreneurship encompasses the activities and processes undertaken to discover, define, and exploit opportunities to enhance social wealth… defined broadly to include economic, societal, health, and environmental aspects of human welfare” (Zahra et al., 2009, p. 522). Opportunities lie in economic, social, societal and environmental multi-dimensional “unsatisfactory” or “suboptimal” equilibria (Martin and Osberg, 2007), and do not rest only on pure market disequilibria.

Social and commercial entrepreneurs differ regarding the type of opportunities they pursue (Saebi et al., 2018): while the latter focus on new needs, large or growing market size in a structurally attractive industry, the former concentrate on serving basic and long-standing needs (Austin et al., 2006). They attempt to solve social issues (Dorado, 2006; Guo et al., 2020), and emerge in a context characterized by social and institutional barriers (Robinson, 2006). They tackle “complex social problems” or even “wicked problems.” These “are defined by their circular causality, persistence, the absence of well-structured alternative solutions, relative lack of room for trial and error learning, constitutive of ‘contradictory certitudes,’ and harboring redistributive implications for entrenched interests” (Rayner, 2006; Dorado and Ventresca, 2013, p. 69).

Social entrepreneurship opportunity identification mobilizes entrepreneurial abilities to shape an entrepreneurial solution to a social problem (Hansen et al., 2011; Yitshaki and Kropp, 2016; Saebi et al., 2018). Opportunities cover multi-dimensional problems that are deeply embedded in difficult social and institutional environments. As a result, SE “benefits accrue primarily to targeted beneficiaries, as opposed to owners” (Alvord et al., 2004; Austin et al., 2006; Mair and Marti, 2006; Miller et al., 2012, p. 618). While commercial entrepreneurship opportunities are for-profit and instrumental to clear the market, in SE, market solutions are used to solve out-of-the-market social problems, and opportunities are instrumental in resolving multi-dimensional disequilibria.

SEOI has sparked some research very recently, focusing on either single antecedents or narrow sets thereof. They identify specific antecedents of SEOI relative to mainstream entrepreneurship (González et al., 2017). We briefly review this literature to classify the readily identified antecedents of SE into the HOKAAs of OI (context, background, social networks, affect, and cognitive process).

#### Contexts

Resources – which are critical to obtain, constitute a central element of context in SEOI (Dorado, 2006; Hockerts, 2006; Murphy and Coombes, 2009). Activism, self-help and philanthropy supply key resources for SE. These take the form of “legitimacy, awareness of social forces, distinct networks, and specialized technical expertise” (Hockerts, 2006, p. 9), as well as either free or low-cost long-term capital, cheap labor, and effective prescribers. This further supports the idea that SEOI requires a convergence of social, economic and environmental inputs (Murphy and Coombes, 2009). This convergence is central because social entrepreneurs identify opportunities where institutional barriers to resources access and entry are high (Robinson, 2006; Dorado and Ventresca, 2013; Guo et al., 2020). SE is distinctive regarding its context where opportunities stand at the crossroads between the market, non-market (society) and institutional spheres.

#### Backgrounds

The entrepreneur’s background largely shapes SEOI (Dorado, 2006; Robinson, 2006). It covers education, work and personal experience, as well as personality traits, attitudes and cognitive properties. Shaw and Carter (2007) describe social entrepreneurs as ethical, entrepreneurial, creative and agenda-setting. Specific personality characteristics stand out. They display “vision, drive and perseverance” (Sharir and Lerner, 2006, p. 7), and “innovativeness, achievement centered, independence, sense of destiny, low risk aversion, tolerance for ambiguity and social value creation” (Brooks, 2009; Nga and Shamuganathan, 2010, p. 263). Finally, they show empathy and moral judgment (Mair and Noboa, 2006; Conway Dato-on and Kalakay, 2016), as well as compassion (Miller et al., 2012). Using the ‘Big Five’ model as a taxonomy of personality traits (Goldberg, 1990), Nga and Shamuganathan (2010) conclude that social entrepreneurs’ key personality traits are agreeableness, conscientiousness and openness. Personality traits, attitudes and cognitive properties blend in with various types of knowledge and experience.

Deeply rooted beliefs stem from experience such as early childhood trauma (i.e., parents’ divorce, depression or suicide, and violence), or from deeply transformative negative or positive experiences (i.e., living abroad and gaining perspective, growing up in a troubled family environment, combating alcohol or drug use, having parents with high levels of social and political engagement, or experiencing early involvement in social issues) (Barendsen and Gardner, 2004). These life events are experience corridors, which “created awareness of and information about particulars areas that shaped opportunity development” (Corner and Ho, 2010, p. 652). These represent extensions of knowledge corridors, defined as information and know-how gained from past work experience and education (Ronstadt, 1988; Shane, 2000). The awareness that SE can respond to the social problem grows from prior knowledge and experience (Hockerts, 2015). Sharir and Lerner (2006) depict this as the need for “personal rehabilitation, search for solutions to individual distress, and obligation to one’s community (e.g., ethnic community) or affiliation (e.g., individuals sharing a problem or common fate)” (p.16). The entrepreneur demonstrates a strong problem-solving culture (Dees, 2012), and can “identify income-generating ventures” without traditional sources of financing (Dorado, 2006, p. 336).

#### Social Networks and Interactions

The social entrepreneur’s social network – characterized by an active community belonging and participation, becomes an inspiration and a source of OI, as well as one of the main places for accessing resources (Hockerts, 2006; Guo et al., 2020). Social support definitely underpins the social feasibility of the venture (Mair and Noboa, 2006; Wang et al., 2019). Qualitative research demonstrates that both social networks size and density display a significant effect on SEOI. Social needs, personal, government, training and consulting, financial, as well as support networks are all relevant to SEOI (Sun and Cai, 2013).

#### Affect

Emphasizing the role of empathy and compassion (Mair and Noboa, 2006; Miller et al., 2012), researchers indirectly point toward the probably significant influence of affect in the process of SEOI (Mair and Marti, 2006; Zahra et al., 2009). In parallel, the nature and intensity of experiences leading to SE also point toward a potentially profound emotional phenomenon (Barendsen and Gardner, 2004; Sharir and Lerner, 2006).

In such cases, the mainstream psychology literature points that emotions display three characteristics:

(1) They have “strongly motivating subjective qualities (…) [like] pleasure or pain”;
(2) They are “initiated by some particular objects or event, real or imagined”;
(3) They “motivate particular kinds of behaviors” (Robinson, 2008, p. 155).

They are therefore shaped by the context, background and network, motivate behaviors and attitudes, and are bound to be an important primary antecedent of OI that will interact with the others.

#### Cognitive Process

A first cognitive model deciphers how compassion can translate into SE thanks to “three mechanisms (integrative thinking, prosocial cost-benefit analysis, and commitment to alleviating others’ suffering)” (Miller et al., 2012, p. 616). A second one underlines how “imprinting or the profound influence of social and historical context [is] constraining the perceptual apparatus of entrepreneurs” (Suddaby et al., 2015, p. 1). It proposes the concept of “reflexivity,” by which individuals can identify or generate opportunities thanks to their abilities to imagine new social realities through a constant process of contrasting and comparing various settings.

### Research Gap and Objectives of the Study

The review of the literature shows that the antecedents of SEOI share common points with the antecedents of mainstream entrepreneurship OI. However, they also display specific characteristics. Exploring these common as well as diverging attributes on a systematic basis aims at building a comprehensive formal conceptual framework for SEOI. In essence, OI results from the HOKAAs: a cognitive process that feeds from the interactions of the entrepreneur’s context, background, affect, and social network.

Our introductory case study shows how a social entrepreneur’s story illustrates the “how” and the “why” of SEOI through a complex dynamic system of antecedents. Through a cognitive process that is weaved into her social entrepreneur story (identifying a gap between a need for specific mental healthcare and existing solutions), Hye-Shin identifies social opportunities that are clearly shaped by her country historical socio-economic context (collective traumas, institutional failure, and lack of resources), her background (education and professional experience in psychiatry), her affect (personal childhood trauma, observer and victim of South Korean collective traumas), and her social networks (institutions, professional national and international networks, former and current patients).

These antecedents fall into the HOKAAs from the mainstream entrepreneurship literature and cover some of the specific ones identified in the SEOI literature (access to resources, activism, experience corridors, childhood trauma, empathy, openness, perseverance, institutional and professional network, imprinting, and reflexivity). Other antecedents presented in the SEOI literature are, however, missing here (philanthropy, personal rehabilitation, personal distress, and financial networks). Finally, some antecedents emerge from the case but are missing from the literature (collective traumas, institutional failure, communities of victims, painful professional experience, and conscientiousness). The discrepancies between a randomly-picked case and the existing literature call for a large-number case investigation for a complete analysis and qualification.

Our main objective is to fill this research gap by sifting through a large number of cases which is “the most meaningful way of understanding social entrepreneurship, both theoretically and empirically” (Pless, 2012, p. 318). In doing so, we systematically explore the antecedents of SEOI and use an inductive research method, which allows potential antecedents to emerge freely through the computerized content analysis of a large textual database of social entrepreneurs’ personal histories.

## Data and Methodology

This qualitative study rests on a computerized content analysis methodology, applied on a database of 2,872 social entrepreneurs’ life stories worldwide. It is conducted inductively to identify the antecedents that most frequently emerge in SEOI.

### Secondary-Source Life Stories for Inductive Theory-Building

The Ashoka community represents the largest multi-dimensional sample of social entrepreneurs including 2,872 fellows (men 61.5% and women 38.5%, excluding changemaker schools and campuses) elected over a 35-year period (1982–2016) in 93 developed as well as developing countries (South America 29.9%; Asia 28.8%; Europe 14.8%; Africa 13.9%; North America 9.2%; Middle East 3.2%; and Oceania 0.2%). They engage in eight topic areas, distributed as follows: civic engagement (18%), development and prosperity (16%), children and youth (15%), human rights and equality (13%), business and social enterprise (12%), environment and sustainability (10%), health and fitness (10%), peace and harmonious relations (5%).

Fellow candidates go through a selection process according to five criteria: (1) The Knockout Test: A New Idea (i.e., innovative solution or approach to a social problem with a lasting change); (2) Creativity (i.e., visionaries capable of engineering their visions into reality); (3) Entrepreneurial Quality (i.e., leaders who see opportunities for change and innovation and devote themselves entirely to it); (4) Social Impact of the Idea (i.e., broad local, national or international system change); (5) Ethical Fiber (i.e., coming across as totally trustworthy).

We collected data from the Ashoka website that publishes fellows’ interviews conducted and transcribed by staff members. The common structured interview guide includes an “introduction” to the project, “the new idea” depicting the innovativeness of the project, “the problem” it is attempting to solve, “the strategy” adopted, and “the person” story. Aiming to identify the antecedents of SEOI, we use “the person” part of the interviews. This section presents the entrepreneur’s own story, offering information about him/herself, detailing his/her background, values, motivations and aspirations (Meyskens et al., 2010) prior and in relation to the set-up of the social enterprise. This material is particularly suitable for conducting an inductive analysis (Patton, 1980; Shaw and Carter, 2007), and inducing new emergent theory from empirical data (Glaser and Strauss, 1967; Eisenhardt, 1989; Gioia et al., 2013).

Such a systematic data collection about successful social entrepreneurs is extremely valuable as it is rarely carried out at the international levels. The consistent multi-round selection process using five criteria provides for a structured and homogeneous sample, while the varying sectors and worldwide source countries ensure an adequate representativity of the multiple contexts and their impact on entrepreneurship (Welter, 2011).

We gather 2,872 written texts (from 800 to 2,500 words each) in the narrative form, created by authors who are neither the research subjects nor the researchers. In the social sciences, the use of secondary data hosted on websites is becoming increasingly important. They are easily and cheaply available, span long time periods and wide scopes, going beyond the usual capabilities of the individual or collective researchers (Vartanian, 2010). These properties allow for replication and variations of their study by all members of the research community. Life stories are durable, representative of real phenomena, and re-analyzable (Krippendorff, 1980). They represent a “narrative truth,” offer insights about the way entrepreneurs liaise and make sense of various events occurring in different periods, and present the “coherence between the events, thoughts, and emotional expressions they choose to introduce” (Yitshaki and Kropp, 2016, p. 6).

We note that our data present a built-in selection toward successful social entrepreneurs. The difficulty of identifying entrepreneurs before their establishment is a classical major stumbling point in entrepreneurship (Davidsson and Wiklund, 2001). We thus acknowledge that our study focuses on the antecedents of OI of successful social entrepreneurs – thereby leaving aside the antecedents of opportunities identified but not developed. We interpret our results accordingly.

Our data also reflect, by nature, socially constructed descriptions of social entrepreneurs and lives interpreted and made textual, rather than true objective facts or accurate scientific truth (Yitshaki and Kropp, 2016). However, what is published as their life story, though partial, is very likely to reflect what they would like to tell about themselves, and what we need to know (Atkinson and Delamont, 2006). The analysis results of stories that would be mere socially acceptable accounts of SE would still be interesting. They would inform us about the characteristics of SE as a social construct, that is the result of the conversations between different economic, political and social institutions including the entrepreneurs themselves.

As a final validity test, we independently check data through a random selection of 20 life stories. We carry out a double-blind analysis to ensure that they actually capture, measure and cover the construct of interest. We then confront our results and confirm data validity. An example of such a case analysis is depicted in the introductory case presentation of social entrepreneur Hye-Shin Chung.

### Inductive Content Analysis of 2,872 Life Stories

As prior knowledge about SEOI is limited, we adopt an inductive approach and draw patterns, themes, and categories directly from the data (Patton, 1980, p. 306). More precisely, we follow a content analysis protocol to explore inductively the antecedents of OI in the context of SE. This technique “is fundamentally empirical in orientation, exploratory, concerned with real phenomena and predictive in intent” (Krippendorff, 1980, p. 9). It represents a trustworthy, unobtrusive, objective, systematic, and replicable technique (Weber, 1990; Cortini and Tria, 2014), and allows valid inferences from text and context, systematic sifting through large volumes of data (Krippendorff, 2004). It can be used for investigating attitudes, interests and values of population groups (Berelson, 1952), or discovering “motivational, psychological or personality characteristics” (Krippendorff, 1980, p. 18). The computerized content analysis of a large number of narrative documents seems therefore extremely well suited to reach the key ideas that form the text skeleton, which contains the essential meaning without the imposition of existing theoretical frameworks. It also ensures a systematic treatment for the analysis and generates higher reliability, precision and speed than human coding (Neuendorf, 2002). The inductive content analysis systematically applies a set of categories to extract, describe and interpret the manifest and latent meaning of textual data (Hsieh and Shannon, 2005). We present the five-step process of our empirical analysis in Table 1.

**TABLE 1:** Five-step process of the content analysis.

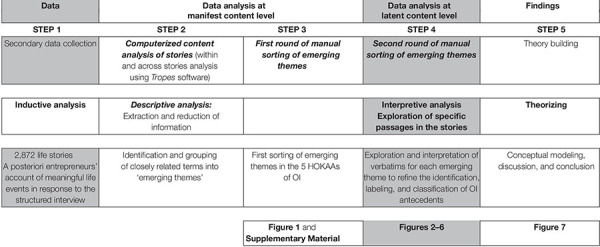

The first step consists in the collection of data. The second step aims at a descriptive account of the reduced and categorized data, exposing the social entrepreneurs’ stories in their own words, namely the manifest or basic level. We enter the full content of the textual data (life stories) into the software for semantic analysis (Tropes), which codes and analyses content using a readily available mainstream dictionary to maintain consistency in criteria. The software walks through the texts to gather terms and list them by frequency. Those terms are then classified both into “Equivalent classes” and “into Reference fields.” “Equivalent classes” group together closely related terms frequently appearing throughout the text (e.g., the terms “wife” and “married woman” are included in the “wife” equivalent class, the terms “marriage,” “wedding,” “marriage proposal,” and “marital status” are part of the “marriage” equivalent class). The “Reference fields” group together “Equivalent classes” to elaborate a representation of the context (e.g., the equivalent classes “wife,” “marriage,” “kin,” “in-laws, etc., form the “family” reference field).

We so obtain a collection of ‘emerging themes’ (or “Reference fields”). These are listed in frequency order: the larger the number of occurrences of reference fields related terms in the text (“wife,” “married woman,” “marriage,” “wedding,” “marriage proposal,” “marital status,” etc.), the higher the frequency of the emerging theme (e.g., “family”). In the third step, where applicable, the authors then manually classify those emerging themes into the five HOKAAs of SEOI obtained from our literature review (context, background, social networks, affect, and cognitive process). As each emerging theme is of interest, regardless of its frequency, and we report them all (see Supplementary Appendix). However, as we aim for generalization across social enterprises, from step four onward, we decide to explore only the first 20 top-frequency themes in further details.

The fourth step consists of the manual exploration of the emerging themes’ related verbatims (themes’ context). Firstly, this allows either confirming or repositioning emerging themes into the five HOKAAs of SEOI. Secondly, within each of the five HOKAAs, we regroup emerging themes into meaningful concepts that constitute the antecedents of SEOI. In the fifth and last step, we identify prominent and repeated relationships between antecedents, closely mirroring reality (Eisenhardt, 1989, p. 547) and escaping the idiosyncratic details (Eisenhardt and Graebner, 2007, p. 30). This step allows building and discussing a theory of SEOI antecedents.

## Results and Discussion

First, we present the themes that emerge from the computerized content analysis of the 2,872 life stories (step 2) and their first-round classification into the five HOKAAs (step 3). Second, we present the antecedents of SEOI that we discover thanks to the second-round sorting and classifying of themes (step 4), and discuss them in the light of the literature. We confirm and further qualify previously identified antecedents, and document and discuss the newly discovered ones. Third, we evidence, explain, and discuss the system of relationships among antecedents (step 5) – the opportunity growing ground.

### Emerging Themes

**FIGURE 1:**
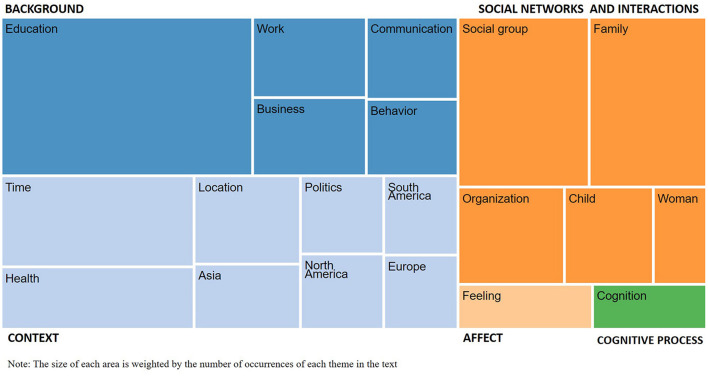
Classification of the top-20 emerging themes into the HOKAAs. The size of each area is weighted by the number of occurrences of each theme in the text.

In Figure 1, we present the first round of manual sorting of the top 20 emerging themes labels into the HOKAAs.

**FIGURE 2:**
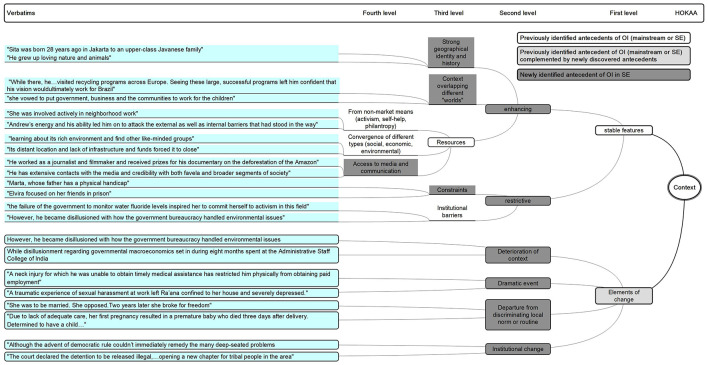
HOKAA ‘Context’: emerging antecedents.

**FIGURE 3:**
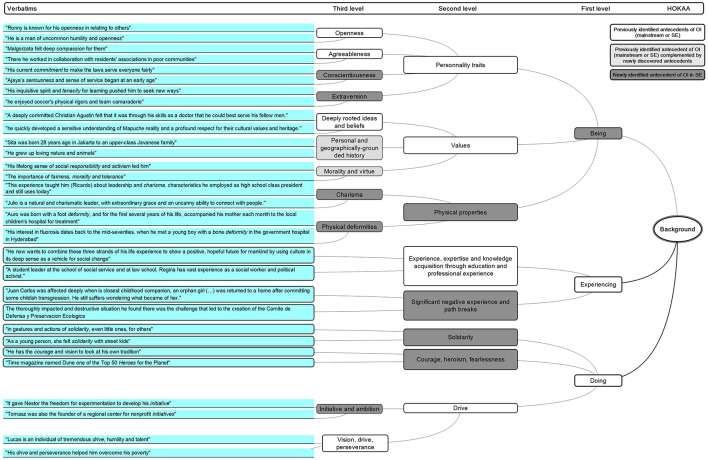
HOKAA ‘Background’: emerging antecedents.

**FIGURE 4:**
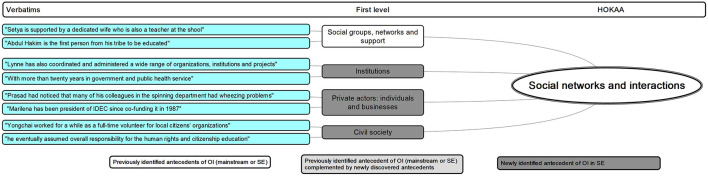
HOKAA ‘Social networks and interactions’: emerging antecedents.

**FIGURE 5:**
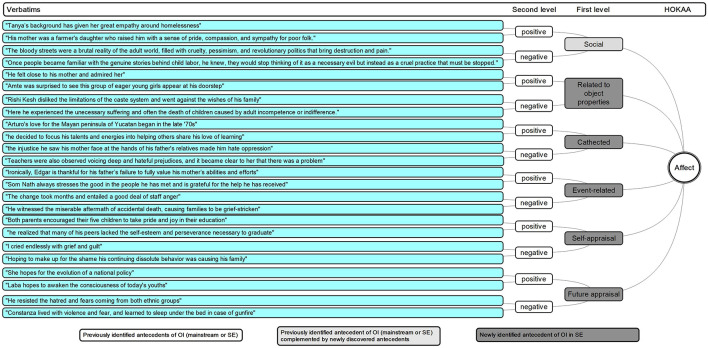
HOKAA ‘Affect’: emerging antecedents.

**FIGURE 6:**
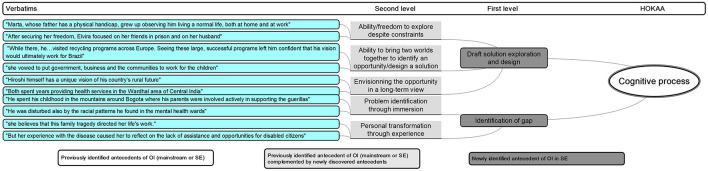
HOKAA ‘Cognitive process’: emerging antecedent.

This first approach shows that 167 themes emerge from the life stories and relate directly to the five HOKAAs identified in the literature (in Supplementary Appendix, see the manual sorting of emerging themes into the five HOKAAs). It appears that each HOKAA contains from one to eight of the top-20 emerging themes. For example, we classify “Time” (ranks 4) in Context; “Education” (rank 1) in Background; “Social group” (rank 2) in Social networks and interactions; “Feeling” (rank 16) in Affect; and “Cognition” (rank 20) in Cognitive process. These results support the use of life stories to examine the antecedents of OI.

The extensive list of themes emerging from a large sample of individuals also shows that the number and variety of potential antecedents of SEOI cover a scope that is wider and more detailed than what the existing entrepreneurship literature suggests.

### A Comprehensive List of Opportunity Antecedents

The second round of manual sorting of emerging themes through the exploration and interpretation of verbatims (step 4) allows us to extract up to four levels of antecedents within each of the five HOKAAs.

#### Contexts

Our analysis suggests four levels of antecedents.

The results validate the existence of two types of first-level antecedents – ‘stable features’ as well as ‘elements of change.’ These confirm that antecedents of SEOI are consistent with the literature on mainstream entrepreneurship identifying “exogenous social, economic, technological and institutional context” (Krueger, 2000; Baron, 2008; Grégoire and Shepherd, 2015), and go beyond the SE literature that solely focuses on ‘stable features.’

Within ‘stable features,’ we verify that ‘institutional barriers’ play a role (Robinson, 2006; Dorado and Ventresca, 2013), and add that ‘constraints’ in various areas of life stand out. These are labeled ‘restrictive stable features.’ We acknowledge that the ‘convergence of various types of resources (social, economic, and environmental)’ are essential (Dorado, 2006; Murphy and Coombes, 2009) while adding the ‘access to media and communication’ as a crucial resource. This is an important element for SE that requires gathering resources by new means (for example, through crowdfunding), and often relies on alternative forms of resources (for example through specific partnerships or networks of volunteers), which require efficient and targeted communication. The idea that resources are often secured through non-market means such as “activism, self-help, philanthropy” (Hockerts, 2006) strongly stands out. The ‘resources’ regroup those three antecedents within the ‘enhancing stable features.’ Within the latter, we show that the context conducive to SEOI often encompasses ‘overlapping different worlds,’ while also presenting ‘strong geographical identity and history.’

More interestingly, we establish that critical ‘elements of change’ are central to the SEOI. These can take the form of a ‘deterioration of the context,’ ‘institutional change,’ ‘dramatic event,’ and a ‘departure from discriminating local norm or routine.’ These types of antecedents had previously been identified in the framework of mainstream entrepreneurship in the form of “specific events” (Baron, 2008) or “precipitating factors” (Krueger, 2000), but had not been scrutinized for the case of SE.

#### Background

Our analysis suggests three levels of antecedents.

Our first-level results match the broad categories, which have previously been categorized in the mainstream entrepreneurship literature (from Ajzen, 1987; Grégoire and Shepherd, 2015). However, we relabel them because we complement and detail the list with antecedents that are specific to SE. Where previous research emphasized general (Dorado, 2006; Robinson, 2006) and/or particular background such as personality traits, prior knowledge and experience, knowledge and experience corridors and socio-historical context (Corner and Ho, 2010; Dees, 2012; Suddaby et al., 2015), we propose three first-level categories of antecedents to SEOI: ‘Being,’ ‘Experiencing,’ and ‘Doing.’

‘Being’ covers ‘personality traits’ among which we validate two of the Big Five (Goldberg, 1990), namely ‘agreeableness’ and ‘openness’ (Nga and Shamuganathan, 2010), and add two others, i.e., ‘conscientiousness’ and ‘extraversion.’ Extending the analysis for ‘agreeableness,’ we identify more precisely “compassion” (Miller et al., 2012).

We additionally find attributes relating to personality traits that qualify how the entrepreneur considers himself: s/he has a sense of ‘worthiness,’ ‘pride and self-esteem,’ ‘humility and modesty,’ as well as ‘respectability and righteousness.’ The social entrepreneur also proves her ‘trustworthiness and responsibility,’ ‘sociality,’ ‘humaneness,’ ‘kindness and goodness,’ as well as ‘courtesy and demeanor, respect, dignity, manners’ which covers and complement the previously identified positive attributes regarding “agreeableness and openness.”

‘Being’ also encompasses ‘Values’ and we confirm ‘deeply rooted ideas and beliefs’ as an antecedent (Barendsen and Gardner, 2004). We add ‘morality and virtue,’ which overlaps the previously identified item of “ethical fiber” (from the Ashoka multi-round selection process and from Drayton, 2002; Shaw and Carter, 2007), “moral judgment” (Mair and Noboa, 2006; Miller et al., 2012; Conway Dato-on and Kalakay, 2016), and ‘personal and geographically-grounded history.’ This also echoes the formerly identified importance of the “social context and history” (Suddaby et al., 2015) in the SE literature. Interestingly, we also find that the social entrepreneur presents noticeable advantageous or disadvantageous physical properties that emerge in ‘attractiveness appearance,’ ‘charisma,’ and ‘physical deformities.’

‘Experiencing’ covers, first, positive experiences that relate mostly to a very wide array of education and professional experience. This further qualifies what authors had identified as “prior knowledge and experience of the social problem” (Dorado, 2006), “experience corridors” (Corner and Ho, 2010), as well as the “encounter of charity and problem-solving cultures” (Dees, 2012). It covers, second, negative experiences that are triggered by the outside world. These match and further detail what previous authors have coined as “early childhood trauma,” “transformative experiences” (Barendsen and Gardner, 2004), “experience of individual distress” (Sharir and Lerner, 2006). These cover ‘significant negative experience and path breaks’ declined into ‘violence and ferocity,’ ‘unrighteousness,’ ‘disrespect and abuse,’ ‘wrongdoings,’ ‘passivity,’ ‘permissiveness,’ ‘individualism and individuality,’ ‘cruelty and evil,’ ‘inattentiveness,’ ‘unsociability,’ ‘exclusion,’ and ‘life path break.’

‘Doing’ shows that social entrepreneurs display a high level of ‘solidarity,’ ‘courage, heroism, fearlessness,’ and ‘drive.’ The latter overlaps the previously identified “vision, drive and perseverance” (Sharir and Lerner, 2006) and add ‘initiative and ambition.’ This “drive” covers various attributes such as ‘discipline,’ ‘resoluteness and self-control, determination, and tenacity,’ and ‘seriousness.’ These, combined with the identified personality traits, confirm the importance of leadership skills (Thompson et al., 2000).

#### Social Networks and Interactions

Our analysis suggests one level of antecedents.

‘Social networks and interactions’ stand out as one of the important HOKAAs of OI defined in the mainstream entrepreneurship literature (from Aldrich and Zimmer, 1986; Grégoire and Shepherd, 2015).

Specific SE first-level antecedents such as ‘social networks’ (Sun and Cai, 2013), ‘social support’ (Mair and Noboa, 2006), and ‘social group,’ strongly emerge from our analysis. The social groups and networks act both as a general support to the social entrepreneurs (Mair and Noboa, 2006), but also as privileged access to essential resources for OI (Hockerts, 2006). Social entrepreneurs rely on their interpersonal and inter-organizational relationships to identify opportunities, to remove some barriers and to gain access to resources held by other actors.

The breadth of our data then allows us to further detail three groups of social actors that seem to be of particular importance to SEOI. These are ‘institutions,’ ‘civil society,’ and ‘private actors’ (mostly individuals in the private sphere such as family and acquaintances, as well as business actors). These actors, as specific resource holders and providers, enhance the possibility to get ideas, gather information and foster OI. More interestingly, we increase the variety of actors of social networks in SEOI. Although the literature recognizes that social entrepreneurs launch social ventures in the context of institutional failure (Robinson, 2006), we evidence the role played by institutions as a social actor part of a network that generates social OI. While previous literature tended to emphasize solely “community” or “affiliation” (Sharir and Lerner, 2006), we show that the social network of the social entrepreneur is, in fact, wide-ranging and encompasses most areas.

#### Affect

Our analysis suggests one level of antecedents.

Our results provide a detailed account – for the case of SE, of the positive and negative emotional antecedents of OI (Baron, 2008; Tang et al., 2012; Foo et al., 2015).

Regarding positive affect, the literature has emphasized the role of “empathy” (Mair and Noboa, 2006; Miller et al., 2012) and “compassion” (Barendsen and Gardner, 2004). We confirm their importance with the emergence in our study of the theme ‘empathy, sympathy, and compassion,’ which we classify in the ‘Social – positive’ first-level category of ‘Affect,’ which also encompasses ‘friendships.’ We equally evidence ‘social – negative’ emotions such as ‘solitariness’ and ‘envies.’ This category bridges with the ‘Social networks and interactions’ HOKAA.

A closely connected series of affects emerges as ‘related to object properties – positive.’ It includes ‘desires, passion,’ and ‘passions,’ which are elements that have been very early on distinguished as one of the characteristics of social entrepreneurs (Bornstein, 1998), as well as ‘admiration and liking,’ ‘enthusiasm,’ and ‘astonishments and surprise.’ ‘Affect’ ‘related to object properties – negative’ also arise in the form of ‘dislikes, alienation, and disapproval.’ This category often links with both the ‘Context’ and ‘Social networks and interactions’ HOKAAs. We also find that ‘cathected – positive’ emotions such as ‘love and affection,’ and ‘cathected – negative’ emotions such as ‘hates’ play a role in the identification of opportunities in SE. These sit at the border between emotions and personality traits (Robinson, 2008), and also connect to ‘Context’ and ‘Social networks and interactions.’

The ‘event-related – negative’ ‘Affect’ reflects the ‘negative elements of change’ of the ‘Context’ and spans ‘difficulty,’ ‘discontentment and frustration,’ ‘suffering, agony, torture, and pain,’ ‘melancholy, sadness, and oppression,’ ‘anger and indignation,’ ‘unpleasantness,’ and ‘culpabilities.’ Without explicitly referring to these, previous literature mentioned “early childhood trauma” and “deeply transformative experiences” (Barendsen and Gardner, 2004). We also uncover two ‘event-related – positive’ ‘Affect’ in the form of ‘pleasure and fun,’ and ‘gratitude,’ possibly linked with the light side of activism, self-help and philanthropy activities (Hockerts, 2006).

The ‘Affect’ regarding ‘self-appraisal’ is merely negative with ‘embarrassments and humiliation,’ reinforced by the negative elements of ‘future appraisal’ ‘Affect’ in the form of ‘skepticism,’ ‘despair, discouragement, and resignation,’ ‘fears and apprehension,’ and ‘nervousness.’ This contradicts some of the ‘Background’ traits of ‘fearlessness’ and could point toward neuroticism as a personality trait (one of the Big Five, according to Goldberg, 1990). These negative elements are, however, in balance with the positive elements of ‘future appraisal’ ‘Affect’ with ‘hope and optimism,’ and ‘expectations.’ Finally, we evidence that the ‘generic positive’ affect in the form of ‘happiness, well-being and satisfaction’ plays a role in SEOI.

The scope and weight of both positive and negative affect definitively demonstrate “strongly motivated subjective qualities.” Affect is initiated by social “objects or events, real or imagined” and generate new social vocation and ventures, as “particular kinds of behaviors” (Robinson, 2008).

#### Cognitive Process

Our analysis identifies two levels of antecedents in the ‘Cognitive process’ (from Kirzner, 1973; Brännback and Carsrud, 2016) that just precedes ‘Opportunity identification.’

We identify two first-level antecedents. First, the ‘identification of a gap’ is possible either by an ‘immersion’ into a specific problem or because of a ‘personal transformation through experience.’ This reflects the process of “imprinting” (Suddaby et al., 2015), which underlines the important effect of the social and historical background of the entrepreneur in the way s/he perceives and analyses situations. Second, the entrepreneur is able of ‘draft solution exploration and design’ regarding the identified problem, because s/he has the ‘ability or freedom to explore despite constraints,’ the ‘ability to bring two worlds together to identify an opportunity/design a solution,’ and to ‘envision the opportunity in a long-term view.’ In those terms, our results explain the ‘how’ “reflexivity” is made possible. It shows how entrepreneurs who are “endowed with the ability to see alternative social and economic arrangements” can consider “the possibility of new and creative social realities” (Suddaby et al., 2015, p. 6).

### The System of Relationships Among Antecedents of Opportunity Identification

**FIGURE 7:**
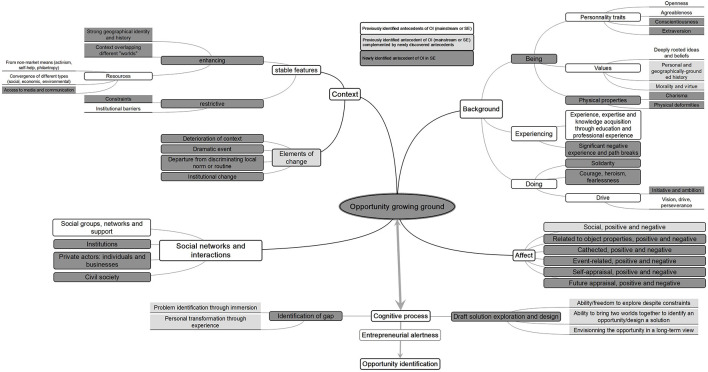
Full model of social entrepreneurship opportunity identification.

We achieve theory building in step 5 of our methodology. It consists in weaving together the five HOKAAs to provide a comprehensive model of SEOI and discuss the links that exist between the different areas. We present the results in Figure 7.

Our large-scale analysis allows the identification of 42 antecedents nested into 17 first-level items grouped into the 5 HOKAAs. Going beyond a simple list, we provide ample evidence that the collection of antecedents of SEOI intricately intertwines into a canvas that represents an ‘opportunity growing ground.’

The context of the social entrepreneur combines stable features regarding access to various resources, a strong geographical identity and history, the encounter of several worlds, all of which condition or are conditioned by her social networks and background. This context also suffers from diverse constraints and institutional barriers that can shape the entrepreneur’s background, her/his experiences, as well as her/his affect specificities. This stable context is at some point hit by elements of change that disrupt this stability, triggering chains of reactions between the various antecedents of OI.

The social entrepreneur’s background presents two broad areas. On one side, it exhibits diverse, strong roots also stemming from the context, a marked sense of morality. The latter can come either from generic (family and/or communities) or more specific (institutions, civil society, private, and business actors) networks, as well as from previous experience (academic and professional background). S/he has a sense of work ethics that emphasizes rigor toward self, and kindness and openness toward others. These qualities are also backed up or fed by significant education and professional experience in related fields. On the other side, s/he quite consistently withstood negative experiences inflicted by other individuals or by the context.

The breadth of affects that emerges from the data is particularly striking and shows that the entrepreneurship literature has underestimated its role. While a certain number of works focus on this particular antecedent, these either treat the role of affect in general or focus on very specific types of affect such as empathy and compassion. We show here a very broad variety of positive and negative affects that play a large role in OI. In essence, the various affects emerge because of the direct or indirect interaction of the entrepreneur with her environment (context, background, or social networks).

Finally, our results show that the cognitive process is indeed one key antecedent of OI. The semantic analysis of verbatims demonstrates that it is a highly specific type of antecedent, which feeds from the context, background, social networks and affects. Through the acquisition and processing of information, the cognitive process consists in the analysis of the opportunity growing ground, and the identification of gaps through immersion or personal transformation. It then pursues by drafting solutions through exploration, bridging worlds, and envisioning the problem in a long-term perspective. The ‘Cognitive process,’ via ‘Entrepreneurial alertness,’ leads finally to the ‘Opportunity identification.’

Each opportunity antecedent has a direct effect on the OI via the opportunity growing ground, and antecedents influence each other as well. We pick some illustrative items from the introductory case of social entrepreneur Hye-Shin. We observe that in a context presenting stable features (older generations often project their psychological status onto younger generations in the form of societal norms and expectations), one or several dramatic events (the Korean collective trauma over the past 60 years and the death of a mother) may occur and trigger a life path change in the background, sometimes in interaction with affect (Having experienced emotional detachment from her mother’s death, becoming a psychiatrist was her singular goal growing up). The background continues to develop thanks to interactions with the further development of both the context (When the financial crisis hit Korea in the late 1990s) and social networks (Her understanding of collective trauma deepened through her work with Dong-won Park… and 20 of his relatives). This illustrates the specific cognitive process of problem recognition through immersion and the drafting of a solution (enable a vast number of ordinary citizens with varying degrees of emotional and psychological needs to access tools and societal support to address their own mental well-being and that of people around them). Our results evidence both the embeddedness of OI in a complex web of antecedents and the endogeneity of the process of OI that is channeled through the interaction of the opportunity growing ground and the cognitive process at play.

## Conclusion

The rapidly developing literature on SE presents the phenomenon as being an integral part of and sharing numerous characteristics with mainstream entrepreneurship. It underlines the specific nature of SE opportunities that represent “wicked” problems. These are embedded in contexts of high institutional and social barriers which resolution translates into benefits accruing to beneficiaries rather than the entrepreneur herself. In parallel, social entrepreneurs are depicted as individuals with specific personality traits and beliefs, who have followed special life paths tainted by deeply marking experiences. To date, however, we know very little about the antecedents that lead individuals to spot and commit to solving complex social issues through entrepreneurship for generally little financial returns. These recent developments call for a further investigation regarding the antecedents of SEOI.

The literature first allows us to pin down five HOKAAs: ‘Context,’ ‘Background,’ ‘Social Networks and Interactions,’ ‘Affect,’ and ‘Cognitive Process.’ We then proceed by way of a five-step content analysis of a large textual database of 2,872 social entrepreneurs’ life stories. The richness of our data and analysis enables us to further detail lower level OI antecedents (42 antecedents nested into 17 first-level items grouped into the 5 HOKAAs) in the specific context of SE.

The ‘Context’ presents ‘Enhancing Stable Features’ in the form of access to tangible and intangible resources, while ‘Restrictive Stable Features’ take the form of various constraints, a large variety of which are institutional. The ‘Elements of Change’ are chiefly negative events except for the occasional facilitating institutional change. The ‘Background’ is composed of the previously identified ‘Experiencing’ and ‘Doing’ elements, which we complement with ‘Being’ elements. The latter encompass new ‘Personality traits’ and ‘Physical Properties’ for social entrepreneurs. Beside the generic ‘Social Groups, Networks and Support,’ we distinguish three categories of important ‘Social Networks and Interactions,’ showing that SE grows within society as a whole. These are ‘Institutions,’ ‘Private Actors: Individuals and Businesses,’ and ‘Civil Society.’ The detailing of ‘Affect’ demonstrates that a vast array of positive and negative emotions – produced by various experiences, precedes SEOI. Finally, we show that the ‘Cognitive Process’ is chiefly composed of two elements: the ‘Identification of Gap’ and ‘Draft Solution Exploration and Design.’

Our detailed analysis allows us to unify the ensemble of antecedents as an ‘Opportunity Growing Ground,’ and build a full model of SEOI based on their interconnections. Indeed, while each antecedent can be studied separately, the full picture allows a deeper understanding of the process. This represents – to the best of our knowledge, the first empirically-backed unification of a theory of OI that demonstrates the consistent presence and interactions of the five HOKAAs.

The context of SE allows us to uncover new antecedents of OI which could open the door to new insights regarding mainstream entrepreneurship. Indeed, this specific context forces us to look at entrepreneurship from a different angle, thereby uncovering previously neglected facets that could be relevant for the study of the general case. Similarly, our results could be transposed to other specific entrepreneurship contexts that share common features with SE. This is, for example, the case for sustainable entrepreneurship, micro-entrepreneurship in developing, transition, or recession economies.

## Entrepreneurial Implications

The first key takeaway for practitioners is that engaging in entrepreneurship goes beyond the simple search for a profit opportunity. It speaks about the identification of an opportunity to solve society’s problems, and this identification is deeply rooted in the entrepreneur’s life story. The context has heavy consequences on the entrepreneur, directly shaping her background, and indirectly influencing it through her social network. These are all elements of life, which is made human by the emotions they trigger. The individual then makes sense of these elements and resolves life’s inconsistencies through entrepreneurship, a form of agency, i.e., a way to operate social change. Such a vision could help entrepreneurs align or realign what they do with who they are.

The second important point that derives from the first is the possibility for all potential entrepreneurs to open up the scope of potential opportunities. This could contribute to the identification of opportunities tackling more ‘difficult’ or ‘challenging’ issues, be it in social or technological terms. This development could help entrepreneurs innovate and build strong competitiveness.

By putting forward the human aspects of entrepreneurship beyond the profit motive, accentuating the need and inclination of entrepreneurs to ‘follow their hearts’, align their entrepreneurial activities with their core values, and therefore live a better life in relationship with themselves, others and their environment, this study hopes to offer a positive social impact. We trust that this will encourage candidate entrepreneurs to ‘make the world a better place,’ because this humane way to envision entrepreneurship can restore and strengthen agency and freedom.

## Limitations and Future Directions

Several limitations of the study could impact the generalizability of our results and therefore, implications for practitioners and future research. These limitations stem mostly from the data used, i.e., a sample of social entrepreneurs from Ashoka Foundation.

First, the thematic investigation consists in analyzing secondary archival profiles of social entrepreneurs rather than primary information about them. In particular, we acknowledge that the coding of the data by a third party could influence the patterns and themes emerging from the data.

Second, the focus on social entrepreneurs does not ensure entirely the generalizability of the results to other types of entrepreneurship. In particular, we have emphasized social entrepreneurship opportunities as being specific in nature, grounded in human development rather than having a commercial purpose. Even if commercial entrepreneurs are also somewhat driven by a social mission, we cannot exclude that they might have varying antecedents and processes of opportunity identification. A study specific to commercial entrepreneurs, or a comparative analysis between social and commercial entrepreneurs could shed some light on this issue.

Our sample of social entrepreneurs presents also a wide heterogeneity in terms of economic and institutional contexts. While our results have the advantage of generalization, it would be very informative to further the investigations and assess the variations in antecedents and processes of opportunity identification along countries’ levels of economic development, social structures and institutional environments.

Last, a comparative study of antecedents and processes of opportunity identification along genders could open up some new avenues of research in the domain of women entrepreneurship.

## Data Availability Statement

A publicly available dataset was analyzed in this study. This data can be accessed here: https://www.ashoka.org/en-us/our-network/ashoka-fellows/search.

## Ethics Statement

Written informed consent was not obtained from the individual(s) for the publication of any potentially identifiable images or data included in this article.

## Author Contributions

Both authors listed have made a substantial, direct and intellectual contribution to the work, and approved it for publication.

## Conflict of Interest

The authors declare that the research was conducted in the absence of any commercial or financial relationships that could be construed as a potential conflict of interest.

## Publisher’s Note

All claims expressed in this article are solely those of the authors and do not necessarily represent those of their affiliated organizations, or those of the publisher, the editors and the reviewers. Any product that may be evaluated in this article, or claim that may be made by its manufacturer, is not guaranteed or endorsed by the publisher.

## Footnotes

1
www.ashoka.org
