# Supplementary material for: A Conceptual System of Antecedents and Processes in Social Entrepreneurship Opportunity Identification
Source: Front Psychol. 2021 Oct 13;12:698892. doi: 10.3389/fpsyg.2021.698892 (PMC8548713; doi:10.3389/fpsyg.2021.698892)
Supplement: Supplementary file 1 [file Table_1.DOCX]

**Appendix: Classification of emerging themes into the HOKAAs, exhaustive list**
